# Pinosylvin Shifts Macrophage Polarization to Support Resolution of Inflammation

**DOI:** 10.3390/molecules26092772

**Published:** 2021-05-08

**Authors:** Konsta Kivimäki, Tiina Leppänen, Mari Hämäläinen, Katriina Vuolteenaho, Eeva Moilanen

**Affiliations:** The Immunopharmacology Research Group, Faculty of Medicine and Health Technology, Tampere University, Tampere University Hospital, 33014 Tampere, Finland; konsta.kivimaki@tuni.fi (K.K.); tiina.leppanen@tuni.fi (T.L.); mari.hamalainen@tuni.fi (M.H.); katriina.vuolteenaho@tuni.fi (K.V.)

**Keywords:** stilbenoid, pinosylvin, macrophage, polarization, interleukin-4 (IL-4), lipopolysaccharide (LPS), M1 phenotype, M2 phenotype, resolution, inflammation

## Abstract

Pinosylvin is a natural stilbenoid found particularly in Scots pine. Stilbenoids are a group of phenolic compounds identified as protective agents against pathogens for many plants. Stilbenoids also possess health-promoting properties in humans; for instance, they are anti-inflammatory through their suppressing action on proinflammatory M1-type macrophage activation. Macrophages respond to environmental changes by polarizing towards proinflammatory M1 phenotype in infection and inflammatory diseases, or towards anti-inflammatory M2 phenotype, mediating resolution of inflammation and repair. In the present study, we investigated the effects of pinosylvin on M2-type macrophage activation, aiming to test the hypothesis that pinosylvin could polarize macrophages from M1 to M2 phenotype to support resolution of inflammation. We used lipopolysaccharide (LPS) to induce M1 phenotype and interleukin-4 (IL-4) to induce M2 phenotype in J774 murine and U937 human macrophages, and we measured expression of M1 and M2-markers. Interestingly, along with inhibiting the expression of M1-type markers, pinosylvin had an enhancing effect on the M2-type activation, shown as an increased expression of arginase-1 (Arg-1) and mannose receptor C type 1 (MRC1) in murine macrophages, and C-C motif chemokine ligands 17 and 26 (CCL17 and CCL26) in human macrophages. In IL-4-treated macrophages, pinosylvin enhanced PPAR-γ expression but had no effect on STAT6 phosphorylation. The results show, for the first time, that pinosylvin shifts macrophage polarization from the pro-inflammatory M1 phenotype towards M2 phenotype, supporting resolution of inflammation and repair.

## 1. Introduction

Macrophages play a major role in the immune system, especially during infection and inflammation, but also in healing and repair. They function as phagocytes that can eradicate micro-organisms and matrix debris. In addition to phagocytosis, macrophages can secrete various cytokines, chemokines, and other pro-inflammatory factors that are involved in eliminating microbes and tumor cells as well as in activating other cells, such as T lymphocytes. In addition, macrophages are able to produce anti-inflammatory cytokines and growth factors to support resolution of inflammation. To simplify, macrophages have been categorized in two principal phenotypes depending on their environment-induced function, either classically or alternatively activated macrophages [1].

Classically activated, also known as proinflammatory M1-type, macrophages functionally participate, especially during inflammation, in the elimination of pathogens by phagocytosis and in the signaling of other inflammatory and tissue cells through secreting pro-inflammatory cytokines and other mediators, such as tumor necrosis factor α (TNF-α), interleukin-1β (IL-1β), interleukin-6 (IL-6), monocyte chemoattractant protein 1 (MCP-1), and nitric oxide (NO), which is produced in high amounts during inflammation by inducible nitric oxide synthase (iNOS) [1]. Proinflammatory M1-type macrophages are usually activated by cytokines secreted by T helper type 1 (Th1) lymphocytes such as interferon-γ (IFN-γ) or by microbial products such as lipopolysaccharide (LPS) [2]. Nuclear factor kappa B (NF-κB) and mitogen-activated protein kinases, particularly C-Jun *N*-terminal kinase (JNK), are significant signaling pathways activated during M1-type polarization [2,3].

Alternatively activated, also known as anti-inflammatory M2-type, macrophages are involved in the resolution of inflammation by scavenging tissue debris and apoptotic cells and by promoting wound healing and tissue regeneration through their pro-angiogenic and pro-fibrotic properties [2]. M2-type macrophages are usually activated by cytokines secreted by T helper type 2 (Th2) cells such as interleukin-4 (IL-4) and interleukin-13 (IL-13) by firstly activating signaling pathways, such as signal transducer and activator of transcription 6 (STAT6) and peroxisome proliferator-activated receptor γ (PPAR-γ). Expression of multiple genes is also specific to M2-type macrophage activation, perhaps the most well-known example being arginase-1 (Arg-1), mannose receptor C type 1 (MRC1), and chitinase-3-like 3 (Ym1) [4].

In the treatment of inflammation, pharmacologically optimal medicine would inhibit and reduce M1-type macrophages, and/or activate and increase M2-type macrophages. Endogenous and exogenous glucocorticoids are regarded as the most effective anti-inflammatory agents, precisely because they can both inhibit proinflammatory M1-type macrophages and enhance the activation of the anti-inflammatory M2-type macrophages [5].

**Figure 1:**
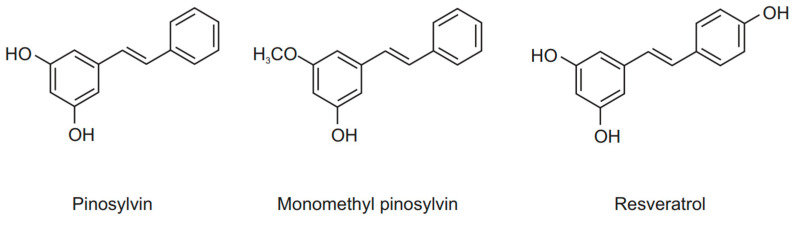
Chemical structures of the stilbenoids investigated in the present study (from left to right): pinosylvin, monomethyl pinosylvin, and resveratrol.

Stilbenoids are a diverse class of phenolic compounds that function as defense mechanism for many plants, and they also have health benefits in humans. Stilbenoids are most commonly found in berries or fruits but also in other types of plants such as ferns and mosses [6,7]. In the present study, we focused on a fairly unexplored stilbenoid, pinosylvin, which can be found particularly in the knots of *Pinus sylvestris*, also known as Scots pine [8]. We also used perhaps the most researched stilbenoid, resveratrol, and the structurally closely related pine stilbenoid monomethyl pinosylvin (Figure 1) as a comparison for the principal effects of pinosylvin.

We have previously reported anti-inflammatory effects of pinosylvin in acute paw inflammation in the mouse, in chondrocytes as well as in classically activated macrophages [9,10,11]. The aim of the present study was to investigate the effects of pinosylvin on the alternative (M2-type) activation of macrophages to verify the hypothesis that pinosylvin has a dual anti-inflammatory effect on macrophages, through enhancing the alternative (M2-type) activation in addition to the earlier known inhibition of the classical (M1-type) macrophage activation.

## 2. Results

### 2.1. Pinosylvin Enhances M2-Type Macrophage Activation in Murine J774 Cells

**Figure 2:**
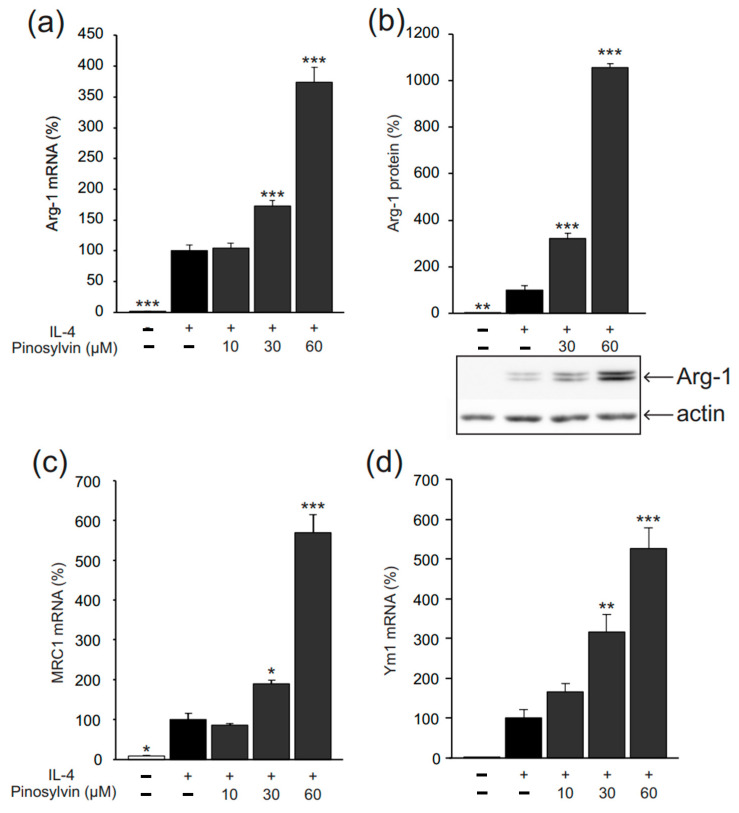
Pinosylvin enhanced IL-4-stimulated expression of markers of alternative (M2-type) macrophage activation in J774 murine macrophages. J774 cells were cultured with IL-4 (10 ng/mL) alone, or together with pinosylvin for 24 h (**a**–**c**) or 8 h (**d**). Expressions of arginase-1 (Arg-1), mannose receptor C type 1 (MRC1), and chitinase-3-like 3 (Ym1) mRNA were measured by quantitative reverse transcriptase polymerase chain reaction (qRT-PCR) and normalized against GAPDH mRNA (**a**,**c**,**d**). Arg-1 protein was measured with Western blot using actin as a loading control (**b**). The results are expressed as mean + SEM, *n* = 6 (**a**,**c**) or *n* = 4 (**b**,**d**). Statistical significance of the results was calculated by one-way ANOVA followed by Bonferroni multiple comparisons test. * = *p* < 0.05, ** = *p* < 0.01, and *** = *p* < 0.001 as compared to cells cultured with IL-4 only, marked as a black bar.

**Figure 3:**
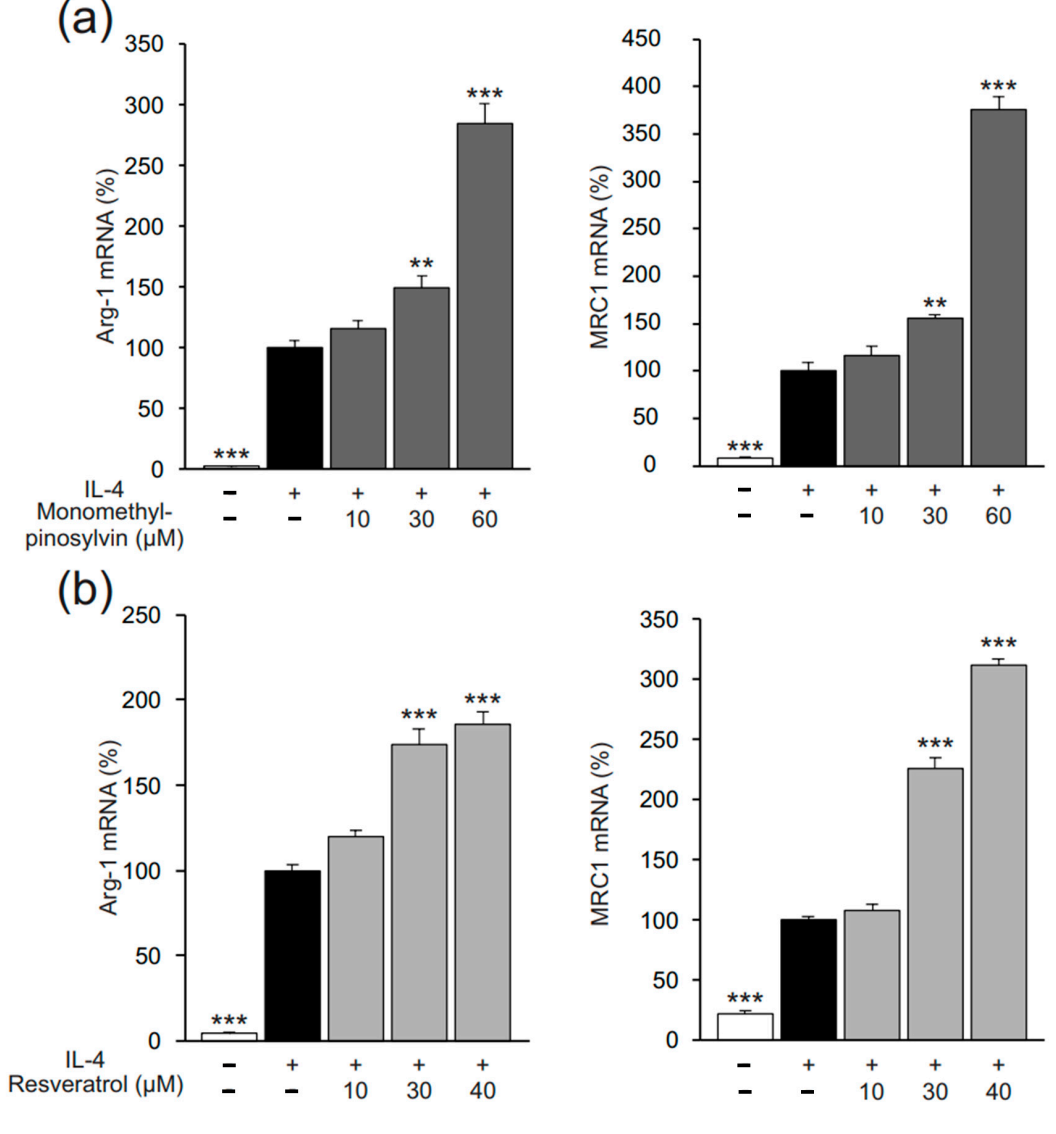
Monomethyl pinosylvin (**a**) and resveratrol (**b**), two natural stilbenoids selected as comparison compounds for pinosylvin, also enhanced IL-4-stimulated expression of markers of alternative (M2-type) macrophage activation in J774 murine macrophages. J774 cells were cultured with IL-4 (10 ng/mL) alone, or together with monomethyl pinosylvin (**a**) or resveratrol (**b**) for 24 h. Expressions of arginase-1 (Arg-1) and mannose receptor C type 1 (MRC1) mRNA were measured by quantitative reverse transcriptase polymerase chain reaction (qRT-PCR) and normalized against GAPDH mRNA. The results are expressed as mean + SEM, *n* = 6 (**a**) or *n* = 4 (**b**). Statistical significance of the results was calculated by one-way ANOVA followed by Bonferroni multiple comparisons test. ** = *p* < 0.01 and *** = *p* < 0.001 as compared to cells cultured with IL-4 only, marked as a black bar.

**Figure 4:**
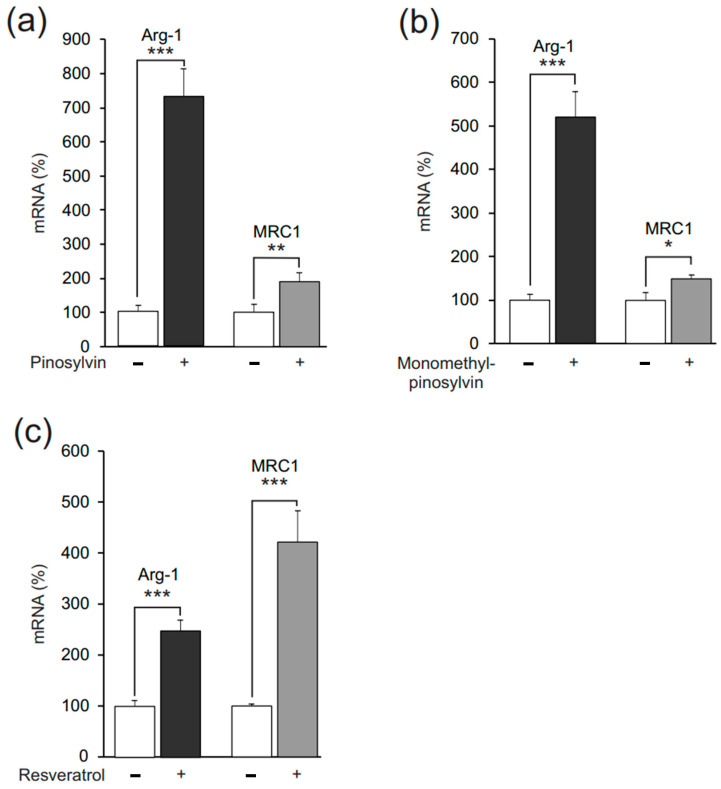
Pinosylvin, monomethyl pinosylvin, and resveratrol enhanced expression of markers of alternative (M2-type) macrophage activation also in the absence of IL-4 in J774 murine macrophages. J774 cells were cultured with pinosylvin (60 µM, **a**), monomethyl pinosylvin (60 µM, **b**), or resveratrol (30 µM, **c**) for 24 h. Expressions of arginase-1 (Arg-1) and mannose receptor C type 1 (MRC1) mRNA were measured by quantitative reverse transcriptase polymerase chain reaction (qRT-PCR) and normalized against GAPDH mRNA. The results are expressed as mean + SEM, *n* = 6 (**a**–**c**). Statistical significance of the results was calculated by *t*-test. * = *p* < 0.05, ** = *p* < 0.01, and *** = *p* < 0.001as compared to cells cultured without stilbenoids, marked as a white bar.

Arg-1, MRC1, and Ym1 were measured as markers of alternative (M2-type) activation in murine J774 macrophages. In the absence of exogenous cytokines, their expression was low, but when IL-4 was added into the culture, it significantly increased their expression. The natural stilbenoid pinosylvin further enhanced M2-type activation, as evidenced by enhanced expression of Arg-1, MRC1, and Ym1 (Figure 2). The effect was shared by two other natural stilbenoids, namely, monomethyl pinosylvin and resveratrol, which elevated expressions of Arg-1 and MRC1 (Figure 3). The stilbenoids increased Arg-1 and MRC1 expression also in the absence of IL-4 (Figure 4).

**Figure 5:**
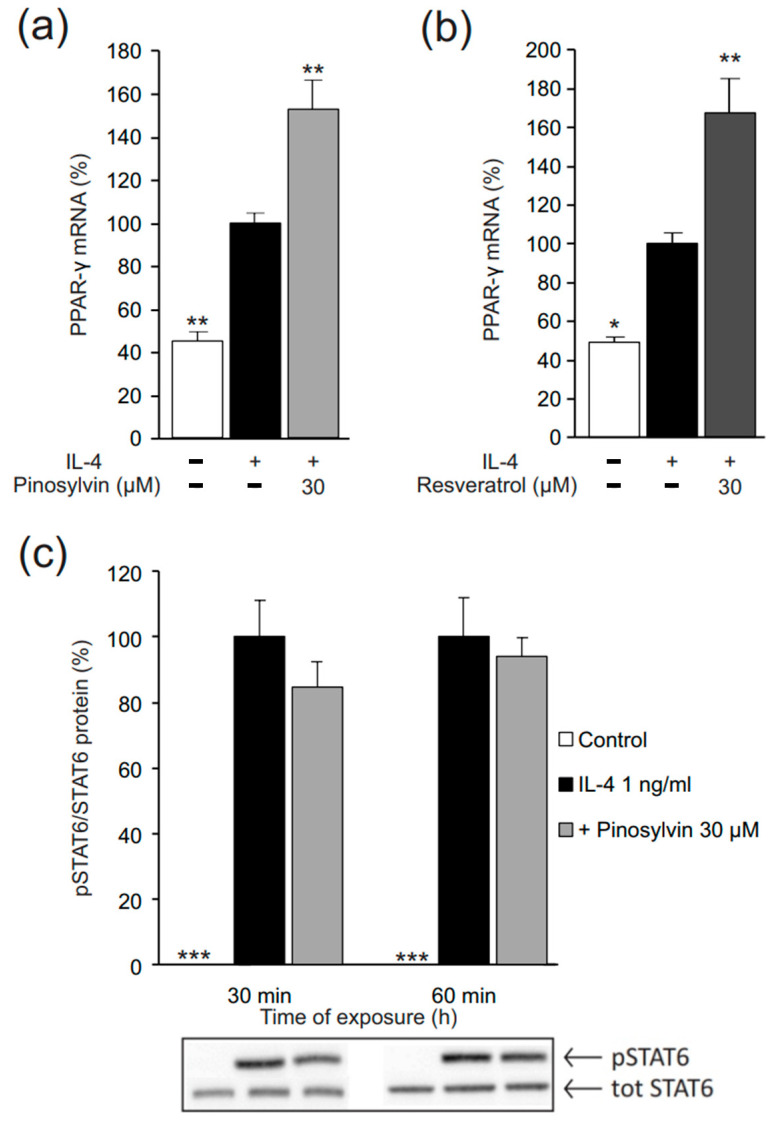
Pinosylvin (**a**) as well as resveratrol (**b**) enhanced IL-4-stimulated peroxisome proliferator-activated receptor γ (PPAR-γ) expression but had no effect on the phosphorylation (i.e., activation) of signal transducer and activator of transcription 6 (STAT6) (**c**) in J774 murine macrophages. J774 cells were cultured with IL-4 (1 ng/mL) alone, or together with pinosylvin or resveratrol for 4 h (**a**,**b**), or for 30 or 60 min as indicated (**c**). Expression of PPAR-γ mRNA was measured by quantitative reverse transcriptase polymerase chain reaction (qRT-PCR) and normalized against GAPDH mRNA. STAT6 phosphorylation was measured with Western blot, and phosphorylated STAT6 was normalized against the total STAT6 protein. The results are expressed as mean + SEM, *n* = 4. Statistical significance of the results was calculated by one-way ANOVA followed by Bonferroni multiple comparisons test. * = *p* < 0.05, ** = *p* < 0.01, and *** = *p* < 0.001 as compared to cells cultured with IL-4 only, marked as a black bar.

PPAR-γ and STAT6 are considered the key signaling mechanisms activated to polarize murine macrophages towards the M2 phenotype. To understand the mechanisms of action of pinosylvin, we measured its effects on PPAR-γ and STAT6. Interestingly, pinosylvin enhanced the expression of PPAR-γ, as did resveratrol, whereas pinosylvin did not alter STAT6 phosphorylation (i.e., activation) (Figure 5).

### 2.2. Pinosylvin Inhibits M1-Type Macrophage Activation in Murine J774 Cells

**Figure 6:**
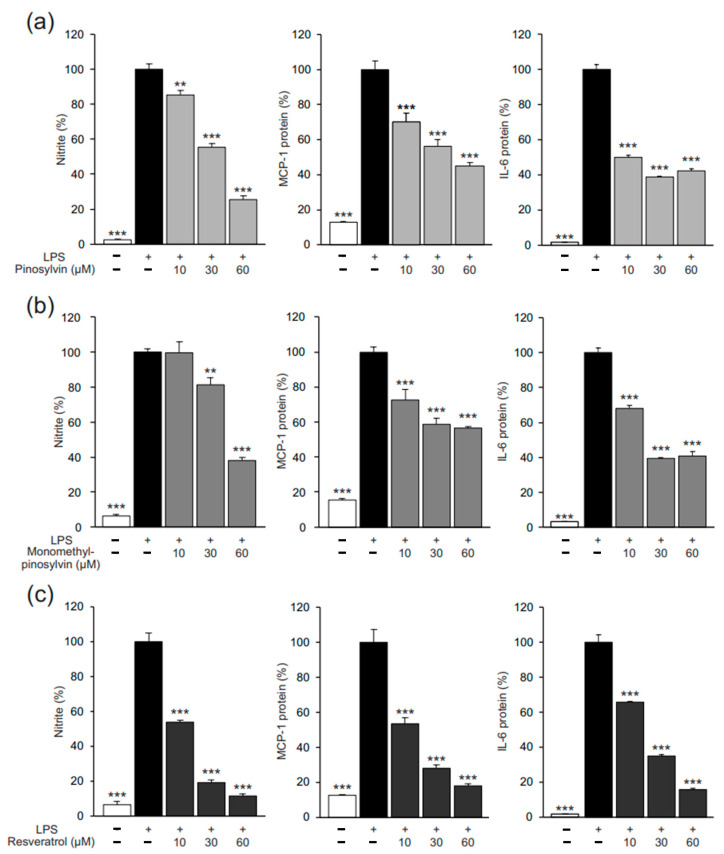
Pinosylvin (**a**), monomethyl pinosylvin (**b**), and resveratrol (**c**) inhibited lipopolysaccharide (LPS)-stimulated expression of markers of classical (M1-type) macrophage activation in J774 murine macrophages. J774 cells were cultured with LPS (10 ng/mL) alone or together with the stilbenoid compounds for 24 h. Nitric oxide (NO) production was measured as its stable metabolite nitrite in the culture medium by the Griess reaction. Monocyte chemoattractant protein 1 (MCP-1) and interleukin-6 (IL-6) concentrations were measured by enzyme-linked immunosorbent assay (ELISA). The results are expressed as mean + SEM, *n* = 6. Statistical significance of the results was calculated by one-way ANOVA followed by Bonferroni multiple comparisons test. ** = *p* < 0.01 and *** = *p* < 0.001 as compared to cells cultured with LPS only, marked as a black bar.

We also examined the effects of pinosylvin on the expression of markers of classical (M1-type) macrophage activation: NO, MCP-1, and IL-6. As expected, pinosylvin inhibited the M1-type activation, which was seen as a decline in all three markers (Figure 6a). The effect was shared by the two other stilbenoids, resveratrol and monomethyl pinosylvin (Figure 6b,c).

**Figure 7:**
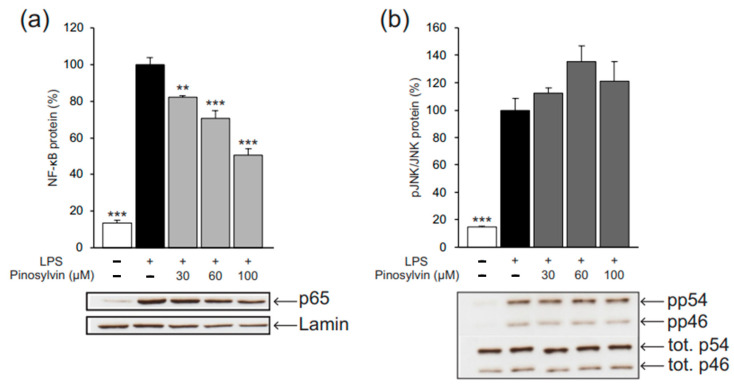
Pinosylvin inhibited lipopolysaccharide (LPS)-stimulated activation of nuclear factor kappa B (NF-κB, **a**), but had no effect on the phosphorylation (i.e., activation) of C-Jun N-terminal kinase (JNK, **b**) in J774 murine macrophages. J774 cells were cultured with LPS (10 ng/mL) alone, or together with pinosylvin for 30 min (**a**); in (**b**) the cells were first preincubated with pinosylvin for 30 min and then stimulated with LPS (10 ng/mL) for one hour. The p65 subunit of NF-κB in the nuclear extract and phosphorylated JNK in the total cell lysate were measured with Western blot. The p65 subunit of NF-κB was normalized against lamin, and the phosphorylated JNK against total JNK. The results are expressed as mean + SEM, *n* = 4. Statistical significance of the results was calculated by one-way ANOVA followed by Bonferroni multiple comparisons test. ** = *p* < 0.01 and *** = *p* < 0.001 as compared to cells cultured with LPS only, marked as a black bar.

In macrophage activation towards the M1 phenotype, the key signaling factors are NF-κB and JNK. We studied the effects of pinosylvin on those markers in order to further understand its mechanism of action in suppressing M1-type activation. We discovered that pinosylvin inhibited NF-κB activation (measured as p65 translocation into the nucleus) but had no effect on JNK phosphorylation (i.e., activation) (Figure 7).

### 2.3. Pinosylvin Induces M2 Polarization Also in Human U937 Macrophages

**Figure 8:**
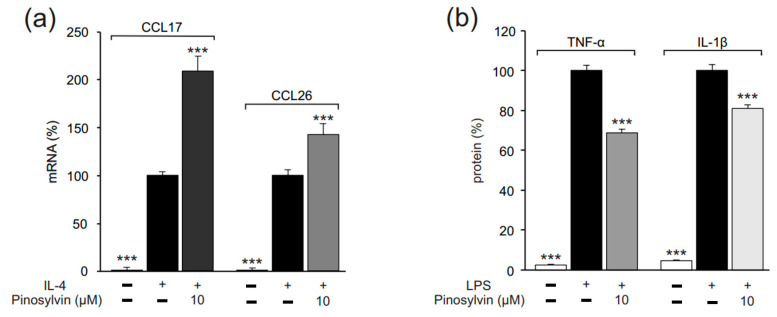
Pinosylvin shifted macrophage polarization towards alternative M2 phenotype in U937 human macrophages. U937 cells were cultured with IL-4 (10 ng/mL, **a**) or LPS (10 ng/mL, **b**) alone, or together with pinosylvin for 24 h. Expressions of C-C motif chemokine ligand 17 (CCL17) and C-C motif chemokine ligand 26 (CCL26) mRNA were measured by quantitative reverse transcriptase polymerase chain reaction (qRT-PCR) and normalized against GAPDH mRNA (**a**). Tumor necrosis factor α (TNF-α) and interleukin-1β (IL-1β) concentrations in the culture media were measured by enzyme-linked immunosorbent assay (ELISA, **b**). The results are expressed as mean + SEM, *n* = 6 (**a**) or *n* = 4 (**b**). Statistical significance of the results was calculated by one-way ANOVA followed by Bonferroni multiple comparisons test. *** = *p* < 0.001 as compared to cells stimulated with IL-4 (**a**) or LPS (**b**) only, marked as a black bar.

After the promising results in the murine J774 macrophages, we wanted to investigate if the observed phenomenon also exists in human macrophages. In human U937 macrophages, we measured C-C motif chemokine ligand 17 (CCL17) and C-C motif chemokine ligand 26 (CCL26) as markers for alternative (M2-type) activation. In the absence of exogenous cytokines, the expression of these M2 macrophage markers was low. When IL-4 was added into the culture, it significantly enhanced the expression of CCL17 and CCL26. Likewise, in the murine macrophages, pinosylvin enhanced the M2-type macrophage activation in human macrophages, which was seen as increased expression of CCL17 and CCL26 (Figure 8a). Pinosylvin also suppressed classical (M1-type) activation in U937 macrophages, as evidenced by its inhibitory effect on LPS-stimulated TNF-α and IL-1β production (Figure 8b). These data indicate that pinosylvin also shifts the macrophage polarization towards alternative (M2) phenotype in human macrophages.

## 3. Discussion

In the present study, we investigated the anti-inflammatory effects of the fairly unknown and inscrutable stilbenoid, pinosylvin, from the aspect of the pleiotropic immune cell macrophage. We hypothesized that pinosylvin could shift macrophage polarization towards the alternative M2 phenotype under inflammatory conditions. The present findings support the hypothesis that pinosylvin not only downregulates classical M1-type activation, but it also enhances alternative M2-type activation of macrophages, which is a novel finding. The results were further supported in the mechanistic studies, where pinosylvin inhibited NF-κB activation and enhanced the expression of PPAR-γ, effects that are implicated in the regulation of macrophage polarization from M1-type to M2-type to support resolution of inflammation and repair. 

In the current study, pinosylvin showed enhancing effects on the expression of the M2 phenotype markers Arg-1, MRC1, and Ym1 in murine J774 macrophages. For comparison, we also measured the effects of two other stilbenoids: perhaps the most researched stilbenoid, resveratrol (found, e.g., in grapes), and the structurally related pine stilbenoid, monomethyl pinosylvin. Interestingly, the effects of pinosylvin were shared by these two compounds, suggesting a group effect of pinosylvin-like stilbenoids. To support this finding, Wang et al. recently reported that resveratrol, and its analogues (other than pinosylvin or monomethyl pinosylvin), enhanced the production of M2-markers (Arg-1, IL-10, and CD163), while inhibiting the production of M1-markers (NO, iNOS, IL-1β, IL-6, and TNFα) in BV-2 mouse microglial macrophages [12]. We were also interested if the effects found in murine macrophages would also be present in human cells. Therefore, we continued by investigating the effects of pinosylvin on the expression of M2-markers in human U937 macrophages. The markers chosen were CCL17 and CCL26, used in previous human macrophage studies [13,14]. As in murine macrophages, the enhancing effect of pinosylvin on M2-markers was found also in human macrophages. 

The anti-inflammatory effects of stilbenoids on classically activated M1-type macrophages have been studied in the past, especially using resveratrol (reviewed in [15]), but there are also a few reports using pinosylvin [9,10]. This effect was replicated in the present study, supporting our hypothesis. In murine J774 macrophages, pinosylvin had a clear inhibiting effect on all three measured M1-markers, NO, MCP-1, and IL-6. Similar inhibitory effects were also observed with resveratrol and monomethyl pinosylvin. We also confirmed our findings in the human U937 macrophage cell line. Pinosylvin had a suppressing effect on the M1-markers TNFα and IL-1β in human macrophages as well, validating our results. 

To further examine the anti-inflammatory properties of pinosylvin, we investigated plausible mechanisms of these effects. Pinosylvin was found to downregulate NF-κB activation (measured as p65 translocation into the nucleus) and to increase the expression of PPAR-γ, the key signaling events polarizing macrophages from the M1-type towards M2-type. To support our findings, resveratrol has been previously reported to have similar effects on these two key signaling mechanisms in related cell types [16,17].

Anti-inflammatory effects of pinosylvin in vivo is still a fairly unexplored subject, although, as mentioned, previous studies have shown its inhibitory effects on M1-type macrophages, and in acute paw inflammation in the mouse [9,10]. In addition, its involvement in other inflammation-related mechanisms or conditions has been studied. In the study by Moilanen and co-workers, pinosylvin was identified as an antagonist of transient receptor potential ankyrin 1 (TRPA1). TRPA1 is an ion channel that functions as a sensor for an array of environmental and endogenous irritants to mediate pain and neurogenic inflammation; pinosylvin was found to inhibit TRPA1-mediated Ca^2+^ influx in vitro and acute paw inflammation in mice [18]. In the study by Lim et al., pinosylvin containing extract from the branches of *Hovenia dulcis* had anti-allergic effects in mast cell-dependent passive cutaneous anaphylaxis (PCA) models in the mouse [19]. In the study by Bauerova and co-workers, methotrexate combined with pinosylvin had a superior effect in adjuvant arthritis in the rat as compared to the treatment with methotrexate alone in decreasing the activity of lipoxygenase, in reducing circulating levels of thiobarbituric acid reactive substances, and in increasing the expression of heme oxygenase-1 in the lungs [20]. Our results may, at least in part, provide mechanisms to understand how pinosylvin mediates these reported anti-inflammatory effects in vivo.

Glucocorticoids, such as dexamethasone, are the most effective anti-inflammatory drugs in clinical medicine and are used in a number of indications. Glucocorticoids enhance the production of anti-inflammatory mediators, reduce the expression of pro-inflammatory factors, and also shift macrophage polarization from the proinflammatory M1 phenotype towards the M2 phenotype to support resolution of inflammation, healing and repair [5]. Glucocorticoids have a wide effect on inflammatory mediators and signaling through their transactivation and transrepression mechanisms, including the suppression of NF-κB activity and the upregulation of PPAR-γ [21,22]. Interestingly, pinosylvin shared some of the effects previously linked to glucocorticoids, supporting their beneficial effects in inflammatory conditions.

In conclusion, the present results show, for the first time, that the pine-derived stilbenoid pinosylvin is able to enhance the alternative (M2-type) activation of macrophages. As pinosylvin also inhibits the classical proinflammatory (M1-type) macrophage activation, it likely shifts macrophage polarization towards the anti-inflammatory phenotype to support resolution of inflammation, healing and repair. This effect seems to be shared by other structurally related stilbenoids and can be exploited in the development of anti-inflammatory treatments. 

## 4. Materials and Methods

### 4.1. Compounds

Pinosylvin and monomethyl pinosylvin were purchased from Carbosynth (Compton, UK), and resveratrol from Tocris Biotechne (Abingdon, UK). All other reagents were from Sigma-Aldrich (St. Louis, MO, USA) unless otherwise stated.

### 4.2. Macrophage Polarization Experiments

Murine J774 macrophages (American Type Culture Collection, Manassas, VA, USA) were cultured at 37 °C in a 5% CO_2_ atmosphere in Dulbecco’s modified Eagle’s medium (DMEM), with Ultraglutamine 1 adjusted to contain 10% heat-inactivated fetal bovine serum (FBS; BioWest, Nuaillé, France), 1 mM sodium pyruvate (Lonza, Verviers, Belgium), 100 U/mL penicillin, 100 μg/mL streptomycin, and 250 ng/mL amphotericin (all purchased from Gibco, Thermo Fisher Scientific, Waltham, MA, USA). Confluent cultures were exposed to fresh culture medium containing the compounds of interest.

Human U937 monocytes (American Type Culture Collection, Manassas, VA, USA) were cultured at 37 °C in a 5% CO_2_ atmosphere in RPMI 1640 medium adjusted to contain 2 mM l-glutamine, 20 mM HEPES, 4.5 g/L glucose (all purchased from Sigma-Aldrich), 1.5 g/L sodium bicarbonate, 1 mM sodium pyruvate, 100 U/mL penicillin, 100 μg/mL streptomycin, and 250 ng/mL amphotericin (all purchased from Gibco) and 10% heat-inactivated FBS (BioWest). Differentiation of the monocytes into macrophages was induced by adding phorbol myristate acetate (PMA, 10 nM) at the time of seeding of the cells on plates for 72 h. Serum starvation (FBS 1%) preceded the experiments for 16 h, and the experiments were started by adding the compounds of interest in fresh culture medium supplemented with 1% FBS and other additives, as stated above.

Cells were seeded on 24-well plates for RNA extraction, preparation of whole-cell lysates for Arg-1, pSTAT6/STAT6, and pJNK/JNK Western blotting, and measurements from cell culture media; on 100 mm Petri dishes for nuclear lysates to measure NF-κB p65 subunit by Western blotting; and on 96-well plates for XTT-test. Activation of the cells into M2-type macrophages was induced with IL-4 (R&D Systems Europe Ltd., Abingdon, UK), and into M1-type with LPS at concentrations and incubation times as indicated. The concentrations of pinosylvin, monomethyl pinosylvin, and resveratrol were chosen based on previous studies [9,11].

Cytotoxicity was assessed by XTT test (Cell Proliferation Kit II, Roche Diagnostics, Mannheim, Germany) by using the same drug concentrations, incubation times, and experimental conditions as the actual experiments. Cell viability was also visually assessed under a microscope after the experiments. None of the tested compounds was observed to induce cytotoxic effects at the concentrations used.

### 4.3. Measurement of mRNA Levels by Quantitative RT-PCR

After the cell culture experiments, GenElute™ Mammalian Total RNA Miniprep Kit (Sigma-Aldrich) was used to extract total RNA. 

Quantitative reverse transcriptase polymerase chain reaction (qRT-PCR) was performed as previously described [23]. The reverse transcription to cDNA was carried out by using TaqMan Reverse Transcription reagents (Applied Biosystems, Foster City, CA, USA) or Maxima First Strand cDNA synthesis kit (Fermentas UAB, Vilnius, Lithuania). qRT-PCR was conducted using TaqMan Universal PCR Master Mix reagent and the Applied Biosystems 7500 Real-Time PCR instrument (Applied Biosystems). The Primer Express^®^ Software (Applied Biosystems) was used for designing and optimizing the probe and primer sequences (Table 1) and appropriate concentrations to detect mouse Arg-1, Ym1, glyceraldehyde 3-phosphate dehydrogenase (GAPDH), and human GAPDH. These primers and probes were purchased from Metabion (Martinsried, Germany). Pre-optimized TaqMan Gene Expression assays were used to detect mouse MRC1 (Mm00485148_m1), PPARγ (Mm01184322_m1), human CCL17 (Hs00171074_m1), and CCL26 (Hs00171146_m1) (Life Technologies Europe BV, Bleiswijk, the Netherlands). The mRNA expression levels were normalized against GAPDH, and after that, the relative mRNA levels were determined using a standard curve method (the genes listed in Table 1) or the ΔΔCt method (the TaqMan Gene Expression assays).

### 4.4. Measurement of Protein Levels by Western Blotting

After the cell culture experiments, whole-cell lysates were prepared for Arg-1, pSTAT6/STAT6, and pJNK/JNK, and nuclear extracts for NF-κB p65 subunit Western blots as previously described [24]. Protein concentrations of these extracts were measured by the Coomassie blue method [25]. SDS-polyacrylamide gel electrophoresis and Western blot analysis were carried out as previously described [26]. Antibodies to detect actin (sc-1615R) and Arg-1 (sc-18351) were purchased from Santa Cruz Biotechnology (Santa Cruz, CA, USA), while phospho-STAT6 (#56554), STAT6 (#9362), phospho-JNK (#9251), JNK (#9252), NF-κB p65 (#3034), and lamin A/C (#2032) antibodies as well as horseradish peroxidase (HRP)-linked goat anti-rabbit antibody (#7074) were all purchased from Cell Signaling Technology (Beverly, MA, USA).

### 4.5. Cytokine and Nitrite Measurements in Cell Culture Media by ELISA and Griess Method

After the cell culture experiments, media were collected and kept at −20 °C until measurements. Enzyme-linked immunosorbent assay (ELISA) was used to measure concentrations of MCP-1, IL-6, TNFα, and IL-1β (R&D Systems Europe Ltd., Abingdon, UK). NO production was measured by the Griess method detecting nitrite, a stable metabolite of NO accumulating into the culture medium [27]. 

### 4.6. Statistical Analysis

Results are shown as mean + standard error of mean (SEM). Unpaired t-test or one-way ANOVA followed by Bonferroni multiple comparisons test reporting adjusted *p*-values were used to assess statistical significance of the results (GraphPad InStat version 3.10 for Windows, Graph-Pad Software Inc., San Diego, CA, USA). *p*-values less than 0.05 were considered statistically significant.

## Figures and Tables

**Table 1:** In-house designed primer and probe sequences used in qRT-PCR.

Primer/Probe		Sequence
mArg-1	forward	5′-TCCAAGCCAAAGTCCTTAGAGATTAT-3′
	reverse	5′-CGTCATACTCTGTTTCTTTAAGTTTTTCC-3′
	probe	5′-CGCCTTTCTCAAAAGGACAGCCTCGA-3′
mYm1	forward	5′-AGTGGGTTGGTTATGACAATGTCA-3′
	reverse	5′-GACCACGGCACCTCCTAAATT-3′
	probe	5′-AGCTTCAAGTTGAAGGCTCAGTGGCTCA-3′
mGAPDH	forward	5′-GCATGGCCTTCCGTGTTC-3′
	reverse	5′-GATGTCATCATACTTGGCAGGTTT-3′
	probe	5′-TCGTGGATCTGACGTGCCGCC-3′
hGAPDH	forward	5′-AAGGTCGGAGTCAACGGATTT-3′
	reverse	5′-GCAACAATATCCACTTTACCAGAGTTAA-3′
	probe	5′-CGCCTGGTCACCAGGGCTGC-3′

## Data Availability

All data are included in the manuscript.
